# Effects of a synbiotic on fecal quality, short-chain fatty acid concentrations, and the microbiome of healthy sled dogs

**DOI:** 10.1186/1746-6148-9-246

**Published:** 2013-12-05

**Authors:** Jason W Gagné, Joseph J Wakshlag, Kenneth W Simpson, Scot E Dowd, Shalini Latchman, Dawn A Brown, Kit Brown, Kelly S Swanson, George C Fahey

**Affiliations:** 1 Department of Clinical Sciences, Cornell University College of Veterinary Medicine, Ithaca, NY, USA; 2 Molecular Research DNA Laboratory, Shallowater, TX, USA; 3 Argyle Kennels, Lowville, NY, USA; 4 Department of Animal Sciences, University of Illinois, Urbana, IL, USA; 5 Vet Med Center 1–120, Cornell University College of Veterinary Medicine, Ithaca, NY 14853, USA

**Keywords:** Sled dog, Diarrhea, Prebiotic, Probiotic, Fecal score

## Abstract

**Background:**

Sled dogs commonly suffer from diarrhea. Although multiple etiologies exist there are limited field studies using synbiotics as a supplement to prevent or treat diarrhea. The objective of this study was to examine alterations in fecal quality, short-chain fatty acids (SCFA), and the fecal microbiome in two groups of training sled dogs fed a synbiotic or microcrystalline cellulose placebo. Twenty clinically healthy training sled dogs randomized into two cohorts (9 synbiotic-fed, 8 placebo-fed) for a 6 week prospective study were examined. Fecal pH and fecal short chain fatty acid (SCFA) concentrations were measured and tag-encoded FLX 16S rDNA amplicon pyrosequencing (bTEFAP) and quantitative real-time PCR were performed at baseline (10 d prior to the study) and after 2 weeks of treatment with a total treatment time of 6 weeks. Fecal scores for all dogs were assessed at baseline and every day for 6 wk after initiation of treatment.

**Results:**

Alterations in the fecal microbiome were observed with a significant rise in Lactobacillaceae in the synbiotic group (P = 0.004) after 2 wk of treatment. A positive correlation was found between Lactobacillaceae and overall butyrate concentration (R = 0.62, p = 0.011) in all dogs. After 5 wk of treatment, there was an improved fecal score and fewer days of diarrhea (Χ^2^ = 5.482, P = 0.019) in the dogs given synbiotic, which coincided with a presumed contagious outbreak shared by all dogs in the study.

**Conclusions:**

Use of this synbiotic results in an increase in presumed beneficial bacterial flora of the host colon which was associated with a decrease in the prevalence of diarrhea in training sled dogs.

## Background

The high prevalence of diarrhea in sled dogs during athletic events has caused researchers to investigate the etiology, with limited results. Previous reports state 7.5% morbidity in long-distance racing sled dogs; however, anecdotally the problem occurs with much greater frequency, and diarrhea represents a leading cause for discontinued racing during distance racing events [1]. *Salmonella* spp. have been suspected to be a contributor to the problem since sled dogs are known to consume raw diets, yet previous studies in Alaskan sled dogs have demonstrated no association between the isolation of *Salmonella* and clinical diarrhea [2]. Other enteropathogens have been implicated including *Clostridium perfringens* and *Clostridium difficile* in companion dogs [3]. A recent study refutes this suspicion as there was no association between the two species of clostridium or their respective toxins and the presence of diarrhea in sled dogs [4]. Viral etiologies have also been investigated with recent work examining canine parvovirus in sled dogs competing in the 2006 Iditarod Trail Race; however the titers did not correlate to clinical manifestations [5]. Additionally, diarrhea has been associated with mental and physical stress which can effect gastrointestinal permeability and motility [6], which is commonly observed during racing conditions and is the prevailing theory surrounding much of the diarrhea observed in racing sled dogs, but has yet to be definitively proven as the cause.

While clinical and field investigations into causes of diarrhea in sled dogs have not revealed a definitive pathogen, dietary alterations are often used to manipulate the content and consistency of feces in companion animals. Previous studies have demonstrated the clinical benefits of probiotics include: inhibiting proliferation of pathogenic bacteria, protecting the intestinal barrier, preventing gut bacterial translocation to blood and distant sites, and modulating immune function [7]. Probiotics have been used in veterinary medicine for a number of years and have been reported to significantly improve fecal scores in dogs with naturally occurring diarrhea [8-10]. They are defined as dietary supplements that contain viable non-pathogenic microorganisms, which are considered to confer health benefits to the host^a^. The typical microorganisms used as probiotics are lactic acid bacteria that are normal inhabitants of the colonic flora and include strains of *Enterococcus, Streptococcus, Bifidobacterium,* and *Lactobacillus* spp*.* These bacteria have a history of safe use in humans and animals, and are approved by the Association of American Feed Control Officials (AAFCO).

Prebiotics (primarily soluble fiber) have been used to alter the gut microbiome and quality of feces in multiple species [11-13]. The proposed advantages of prebiotics are that they are metabolized by the selected bacterial species of the colon [14]. A variety of compounds may act as prebiotics, but the great majority of them come in the form of fiber and are typically oligosaccharides. Fermentation by the colonic bacteria of these compounds (specifically the *Lactobacillus* genera) will generate SCFAs including acetate, propionate, and butyrate that may act as a preferred fuel source for the colonocytes [15]. Some prebiotics (galactooligosaccharide) appear to exert a direct antimicrobial effect by adhering to the binding sites on the enterocyte surface and blocking the adhesion of pathogenic bacteria to intestinal epithelial cells [16,17].

A synbiotic is a combination of a probiotic(s) and prebiotic(s). Synbiotics are designed not only to introduce beneficial bacterial populations, but also to promote proliferation of autochthonous-specific strains in the intestinal tract [18]. To date, little research has been performed using synbiotics in companion animals. The objectives of the current study were to feed a synbiotic and a placebo to two groups of sled dogs being exposed to identical dietary substrate and environmental conditions during peak training for competitive racing. We hypothesized that this would result in an increased amount of SCFA production that would increase colonic health, thereby resulting in improved fecal quality and potentially decrease the episodes of diarrhea in training sled dogs.

## Methods

### Study population

This study was approved by the Cornell University Institutional Animal Care and Use Committee. All dogs enrolled in the study underwent a physical examination, complete blood count, and serum biochemistry panel. Twenty healthy, normal Alaskan Husky dogs between 22–26 kg were enrolled in the study and any dogs with elevated white blood cell counts or signs of organ dysfunction were excluded. Dogs were between the ages of 2 and 6 yr old, including 10 intact males and 10 intact females. All dogs were housed in individual outdoor kennels, with all dogs being kept on a clay dirt surface that was cleaned twice daily.

Twenty dogs were randomly assigned to one of two groups by designating one dog from each gender matched pair to either treatment or placebo groups based on a coin flip. Dogs were kenneled individually, but were not segregated to treatment groups during training runs or travel, allowing dog interactions between the two groups. All dogs trained 3–4 times a week, running between 6–12 miles in upstate in the Lowville, New York, USA area. Dogs were in training throughout the entire time of the study and traveled to two races during the study. The dogs traveled to Kalkaska, MI, USA on the weekend of January 16th and 17th just prior to initiation of the study and then on February 14th and 15th to Mannsville, NY, USA. Daily fecal scoring for each dog was performed by the same author (DAB) beginning on January 18th for 10 d to establish a baseline fecal score. Treatment was blinded whereby dogs received either 5 g of synbiotic^b^ or 5 g of placebo^b^ from pails labeled “1” and “2” respectively. The powdered synbiotic or placebo was mixed into the dogs daily feed between 9 and 11 a.m. every morning for 6 wk beginning on January 28th, 2010.

#### Food and supplement analysis

All dogs were fed the same diet. The ration contained a mix of two canine dry pet products with a 5:1 volume ratio of the kibble^c^ to the dry powder^d^ (Additional file 1), mixed with less than 20% volume/volume of ground beef. The feed mixture was analyzed for crude protein, crude fat, moisture, and crude fiber by Dairy One Analytical Services (Ithaca, NY). Upon analysis, the ration was 46% crude protein, 32% crude fat, 7% ash, 3% crude fiber on a dry matter basis and approximately 5.2 kcal ME/g. Samples of the raw meat were also swab cultured and sent to Cornell University Animal Health Diagnostic Center Laboratory for anaerobic and aerobic culture of selected microbial organisms for *Salmonella*, *Campylobacter,* and *E. coli*. A standard aerobic microbial culture and enrichment of the synbiotic and placebo was performed by the Cornell University Diagnostic Laboratory to ensure viability of organisms, before and immediately after the study. The synbiotic contained *Enterococcus faecium* SF68 (56.7 mg/g; 5.67 × 10^8^ CFU/g), *Bacillus coagulans* (2.5 mg/g; 3.75 × 10^7^ CFU/g), *Lactobacillus acidophilus* (14.4 mg/g; 7.2 × 10^8^ CFU/g), fructooligosaccharides (400 mg/g), mannanoligosaccharides (80 mg/g), Vitamin B_1_ (2.5 mg/g), Vitamin B_2_ (0.8 mg/g), Vitamin B_3_ (19.2 mg/g), Vitamin B_6_ (0.8 mg/g), brewer’s yeast (80 mg/g), soy lecithin (30 mg/g), magnesium stearate (10 mg/g), microcrystalline cellulose (266 mg/g), mono-and diglyceraldehyde (30 mg/g), and silica dioxide (7 mg/g). The placebo contained microcrystalline cellulose (629 mg/g), brewer’s yeast (190 mg/g), soy lecithin (71 mg/g), magnesium stearate (24 mg/g), mono-and diglyceraldehyde (71 mg/g), and silica dioxide (16 mg/g).

#### Fecal collection and scoring

Daily fecal scoring was performed (DAB) as an average of feces observed on a daily basis during kennel clean up and recorded. All feces were collected from every dog over a 2 d period on days 9 and 10 of fecal scoring and the mean of each dog was determined (baseline), prior to initiation of the supplementation with feces being collected within 5 min of defecation and immediately frozen at -20°C. Feces were also collected and immediately frozen from all dogs over a 2 d period 2 wk after initiation of the synbiotic or placebo treatments. All fecal samples were transported frozen to the laboratory within 2 wk of collection for pH testing and selective culturing utilizing culture swabs with immediate plate streaking for *Salmonella* spp. and *Campylobacter* spp. Giardia testing was performed on all fecal samples using SNAP ELISA kits. Fecal quality was assessed for every defecation by using a 5-point visual scale, ranging from 1 (hard and dry feces) to 5 (liquid diarrhea) [19]. A score of 2 represented a well-formed stool that was easy to collect but was not too dry; this was considered optimum. A daily score was tallied based on the average score of all feces for that day. Due to the propensity for contagious or other diarrhea outbreaks occurring after initiation of training and racing, the daily mean fecal scores from Sunday to Sunday were averaged and presented as an average fecal score for each week per group. The weekly average fecal scores were then subtracted from the average fecal score before the study began (baseline score) to provide an average change in score for each dog (with positive trends being worse), as the fecal scores at baseline were considered to be normal fecal consistency for that 10 day period according to the kennel owner (DAB). Fecal scoring was further categorized into normal (scores 1–3), or diarrhea (4 and 5) during weeks where significant differences were detected between groups to define whether diarrhea was being detected at a greater rate, defined as days of diarrhea within each week for each dog.

#### Fecal pH and SCFA analysis

Fecal pH and SCFA was determined for each dog at baseline and after 2 wk of treatment. Fecal pH was performed by taking 2 g of the thawed fecal samples and mixing with 1 part water to 1 part feces using a Mettler Toledo InLab® Expert Pro PH meter. Fecal SCFA were quantified by adding a 1 g portion of a fecal sample to 4 mL of water and 1 mL of 25% m-phosphoric acid, mixed well, and allowed to precipitate for 30 min, then centrifuged at 20,000× *g* for 20 min. The supernatant was decanted and frozen at −80°C in microfuge tubes. After freezing, the supernatant was thawed and centrifuged in microfuge tubes at 10,000× *g* for 10 min. Concentrations of acetate, propionate, and butyrate were determined in the supernatant using a Hewlett-Packard 5890A series II gas chromatograph (Palo Alto, CA) and a glass column (180 cm × 4 mm id) packed with 10% SP-1200/1% H_3_PO_4_ on 80/100+ mesh Chromosorb WAW (Supelco Inc., Bellefonte, PA). Oven temperature, detector temperature, and injector temperature were 125, 175, and 180°C. Short-chain fatty acid concentration values also were corrected for blank tube production of SCFA. The supernatants were analyzed using the spectrophotometric method described by Barker and Summerson [20]. All samples were run in duplicate and an error no greater than 5% was considered acceptable.

#### Fecal DNA extraction and bTEFAP

Samples were homogenously mixed after initial thawing and 5 g of feces were shipped on ice overnight to the Research and Testing Laboratory (Lubbock, TX, USA) for Tag-encoded FLX 16S rDNA amplicon pyrosequencing (bTEFAP). Fecal samples were homogenized and 200 mg aseptically suspended in 500 μl RLT buffer (Qiagen, Valencia, CA, USA) (with β-mercaptoethanol). A sterile 5 mm steel bead (Qiagen, Valencia, CA, USA) and 500 μl volume of sterile 0.1 mm glass beads (Scientific Industries, Inc., NY, USA) were added for complete bacterial lyses in a Qiagen TissueLyser (Qiagen, Valencia, CA, USA), and run at 30 Hz for 5 min. Samples were centrifuged and 100 μl of 100% ethanol added to a 100 μl aliquot of the sample supernatant. This mixture was added to a spin column, and DNA recovery protocols were followed as instructed in the Qiagen DNA Stool Kit (Qiagen, Valencia, CA, USA) starting at step 5 of the protocol. DNA was eluted from the column with 50 μl water and samples were diluted accordingly to a final nominal concentration of 100 ng/μl. DNA samples were quantified using a Nanodrop spectrophotometer (Nyxor Biotech, Paris, France). Once the DNA was isolated the bTEFAP methodology was instituted to examine the universal bacterial diversity within the feces. A 100 ng (1 μl) aliquot of each samples’ DNA was used for a 50 μl PCR reaction. The 16S universal Eubacterial primers 530 F (5′-GTG CCA GCM GCN GCG G) and 1100R (5′-GGG TTN CGN TCG TTG) were used for amplifying the 600 bp region of 16S rRNA genes. HotStarTaq Plus Master Mix Kit (Qiagen, Valencia, CA, USA) was used for PCR under the following conditions: 94°C for 3 min followed by 32 cycles of 94°C for 30 sec; 60°C for 40 sec and 72°C for 1 min; and a final elongation step at 72°C for 5 min. A secondary PCR was performed for FLX (Roche, Nutley, NJ, USA) amplicon sequencing under the same condition by using designed special fusion primers with different tag sequences as: LinkerA-Tags-530 F and LinkerB-1100R. The use of a secondary PCR prevents amplification of any potential bias that might be caused by inclusion of tag and linkers during initial template amplification reactions. After secondary PCR, all amplicon products from different samples were mixed in equal volumes, and purified using Agencourt Ampure beads (Agencourt Bioscience Corporation, Danvers, MA, USA).

### Fecal DNA extraction and quantitative real-time PCR

To confirm bTEFAP results and to examine species that were not included in bTEFAP, quantitative changes in fecal abundance of *Bifidobacteria, Lactobacillus, and Enterococcus* spp. in all dogs from baseline to 2 wk after treatment was assessed. Bacterial DNA was extracted using a PowerSoil DNA isolation kit (Mo Bio Laboratories, Carlsbad, CA, USA) based on the manufacturer′s protocol. Fecal DNA was quantified using a GE NanoVue spectrophotometer (GE Healthcare, Buckinghamshire, UK). Extracted DNA was diluted to 5 ng/μl. Quantitative real-time PCR using bacterial universal and genus-specific primers to generate standard curves and data for each genera (*Lactobacillus jonhsonii-* ATCC - 53672*, Bifidobacterium animalis* ATCC −700541*, Enteroccus faecium –*ATCC BAA- 2320) were performed using SYBR Green-based assays (Applied Biosystems, Carlsbad, CA) and protocols as described in Mazcorro, et al. [21]. Using a commercial real-time PCR thermocycler (StepOnePlus; Applied Biosystems, Carlsbad, CA, USA).

### Statistical analysis

After examining quantile plots and Shapiro-Wilk testing, it was determined that much of the microbiota and SCFA data were not normally distributed; hence Wilcoxon Signed Rank testing was used for statistical analysis before and after treatment for pH, fecal scoring, percent microbial flora change, and SCFA concentrations. Chi-square analysis was performed to compare days of diarrhea between the placebo and synbiotic groups during each week of the trial. A linear regression analysis was performed for each significant family of bacteria to each SCFA and total SCFA at baseline and 2 wk after treatment for all dogs. An operational taxonomic unit was considered significant if it populated more than 1% of the entire flora in a fecal sample, and thus was included in the analysis of percentage change associated with treatment as well as assessed in linear regression analysis against SCFA production. For all statistical analyses the α was set at P ≤ 0.05. All statistical analyses were performed using SigmaPlot 11.0^e^.

## Results

### Dogs and supplement analysis

Three dogs were excluded from the study due to acute injuries during training resulting in 17 dogs completing the study (9 dogs receiving synbiotic and 8 dogs receiving placebo). Anaerobic and aerobic microbial culturing of the raw meat resulted in no organism growth (< 100 CFU/50 cm^2^). Initial microbial analysis showed viable organisms in the synbiotic at baseline, before implementing treatment. At the end of the trial, only *Enterococcus* spp. could be cultured from the synbiotic supplement.

### Fecal pathogens, pH, scores, and SCFA analyses

**Figure 1:**
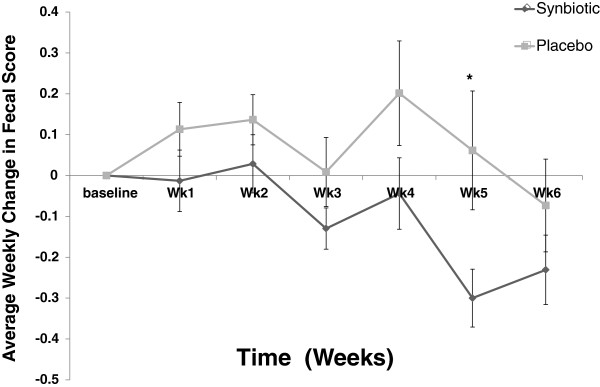
**Mean weekly change and standard deviation in group fecal scores from baseline for 6 weeks after initiation of placebo or synbiotic treatment.** Initial mean score for placebo was 2.91 and 3.06 for synbiotic. *indicates a p < 0.05 for the indicated time point.

No pathogens were isolated from aerobic microbial plate streaking for *Salmonella* spp. or *Campylobacter* spp.. Giardia SNAP tests revealed only one positive in the probiotic group in January. No significant difference in fecal pH was observed between the two groups (P = 0.33). Initial average fecal scores for placebo and synbiotic groups were 2.91 ± 0.22 and 3.08 ± 0.24 respectively, out of the 5 point scale. As a comparison, the mean differences (and standard deviation) from baseline fecal score to the end of each week is depicted in Figure 1. A statistically significant difference between the two groups (P = 0.02) was observed between weeks 4 and 5 of treatment. When examined as total days of diarrhea between the two groups, the synbiotic group showed significantly fewer days of diarrhea than the placebo group (synbiotic group - 6 d; total of 3 dogs; Cider, Marten, Thistle; average duration 2 days); placebo group (17 d total of 4 dogs; Sunny, Raven, Nimbus, Booty; average duration 4 days; Χ^2^ = 5.482, P = 0.019) which coincided with a presumed contagious outbreak in the kennel. No change (P > 0.05) in acetate, propionate, or butyrate concentrations occurred within the synbiotic or placebo groups or between the two groups (data not shown). A linear regression analysis comparing significant microbial families (any family comprising 1% or more of entire fecal microbiome) to individual and total SCFA in feces revealed a positive correlation between Lactobacillaceae and overall butyrate concentration (R = 0.62, P = 0.011).

### Fecal microbiota

**Figure 2:**
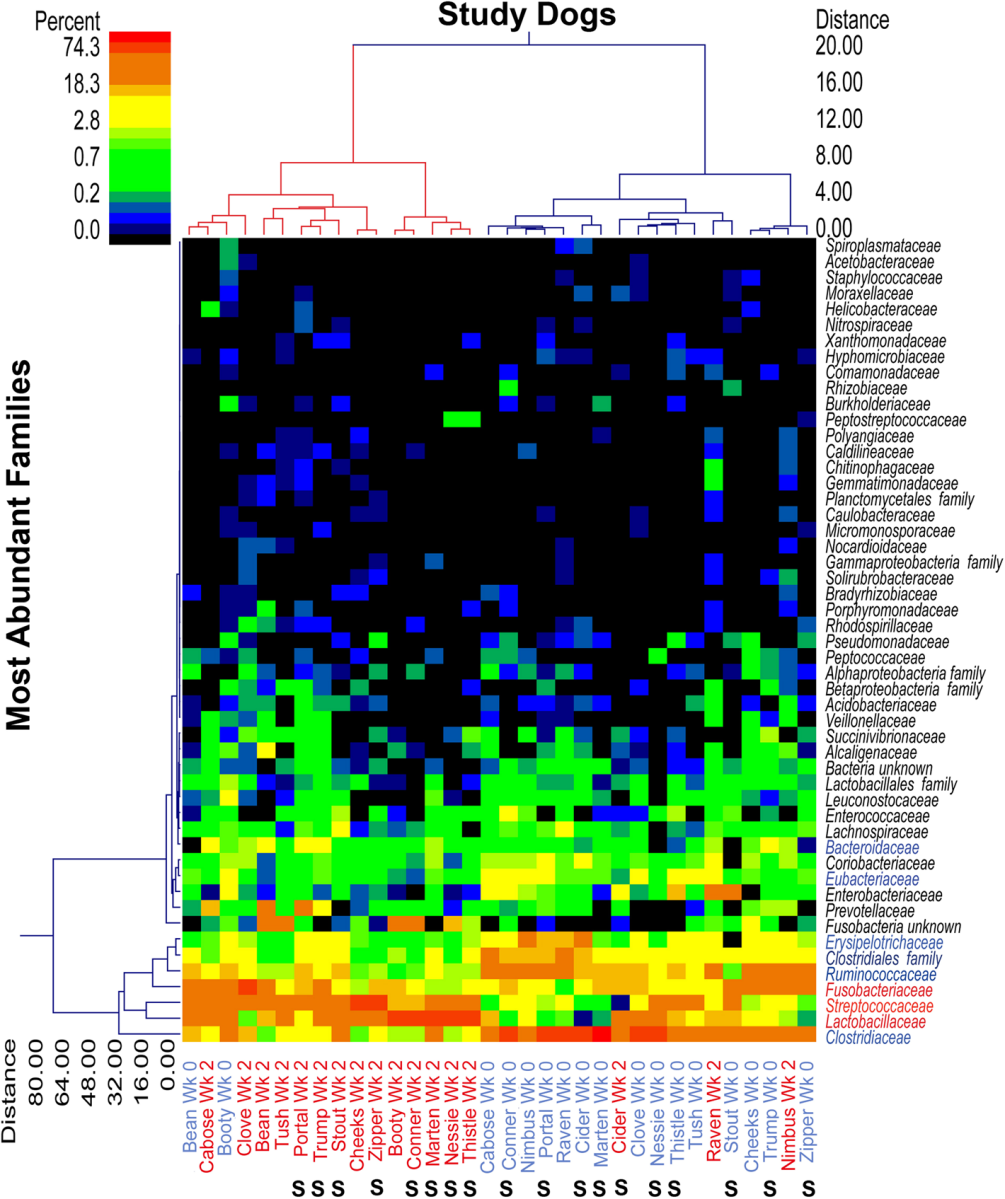
**Dual hierarchical clustering dendrogram of the 50 most abundant bacterial families of all dogs (n = 17) at the January and February samplings showing grouping based on time of sampling and clustering of dogs on the synbiotic (designated with S below their names) in the February sampling that is not observed in the January sampling (S designated dogs dispersed along the time period).** This dendrogram is based on the Wards clustering and Manhattan distance methods. The heatmap depicts the relative percentage of each bacterial family for each sample. The relative distance scale for the left y-axis is provided in the lower left corner of the figure. The color scale for the heatmap is shown in the upper left corner of the figure.

There were no significant differences in fecal bacterial populations between the synbiotic and placebo groups before treatment. Within the group receiving synbiotic (Table 1), bTEFAP results from fecal samples after 2 wk of treatment showed an increase in Lactobacillaceae (P = 0.004), and decreased Clostridiaceae (P = 0.004), Erysipelotrichaceae (P = 0.039), and Eubacteriaceae (P = 0.039) from baseline. Within the group receiving placebo (Table 2), fecal samples after 2 wk of treatment showed decreases in Clostridiaceae (P = 0.008), Ruminococcaceae (P = 0.008), Erysipelotrichaceae (P = 0.008), and Eubacteriaceae (P = 0.039) from baseline. When comparing percentage changes (of the significant microbial families represented in Tables 1 and 2) from baseline to after 2 wk of treatment between the two groups, the only microbial family demonstrating statistical significance was Lactobacillaceae (P = 0.039). A dual hierarchical clustering dendrogram of the 50 most abundant bacterial families created from the bTEFAP data indicated as a definite stratification into two microbiome populations based on a time (January vs. February) with a few outliers at each time point. There also appears to be a tighter clustering of dogs in February that were synbiotic-fed (as indicated by S in the February group) except for a single outlying dog (Cider), suggesting that the microbiome alteration segregates on both time and treatment groups (Figure 2).

**Table 1:** Percent (medians and ranges) of microbial families comprising 1% or more of the microbiota in the feces of synbiotic-fed dogs at baseline and after 2 wk of treatment based on bTEFAP analysis

**Microbial family**	**Synbiotic group (n = 9)** **% at baseline**	**Synbiotic group (n = 9)** **% after 2 wk of treatment**	**Synbiotic group (n = 9)** **P-value**
Lactobacillaceae	0.38 (0.02-21.18)	35.31 (6.66-70.23)	0.004
Clostridiaceae	48.13 (19.91-74.30)	5.28 (1.19-40.29)	0.004
Erysipelotrichaceae	3.45 (0.00-12.70)	1.02 (0.12-2.97)	0.039
Eubacteriaceae	0.88 (0.15-4.27)	0.28 (0.05-0.89)	0.039
Streptococcaceae	2.15 (0.17-14.34)	17.96 (0.02-56.29)	0.055
Ruminococcaceae	9.71 (0.88-22.94)	3.73 (0.91-9.74)	0.055
Bacteroidaceae	0.07 (0.00-1.85)	0.27 (0.00-3.83)	0.055
Alcaligenaceae	0.02 (0.00-0.27)	0.02 (0.00-0.18)	0.578
Leuconostocaceae	0.33 (0.00-0.68)	0.08 (0.00-0.82)	0.383
Prevotellaceae	0.01 (0.00-1.20)	0.26 (0.00-18.92)	0.078
Lachnospiraceae	0.54 (0.00-1.08)	0.12 (0.01-2.64)	0.203
Enterobacteriaceae	0.60 (0.04-14.99)	0.09 (0.00-1.91)	0.074
Coriobacteriaceae	0.66 (0.00-3.04)	0.58 (0.07-1.67)	0.496
Fusobacteriaceae	0.07 (0.00-2.23)	0.15 (0.00-27.86)	0.203
Succinivibrionaceae	0.20 (0.00-1.29)	0.07 (0.00-0.86)	0.641
Enterococcaceae	0.27 (0.00-2.99)	0.35 (0.01-1.29)	0.911

**Table 2:** Percent (medians and ranges) of microbial families comprising 1% or more of the microbiota in the feces of placebo-fed dogs at baseline and after 2 wk of treatment based on bTEFAP analysis

**Microbial family**	**Placebo group (n = 8)** **% at baseline**	**Placebo group (n = 8)** **% after 2 wk of treatment**	**Placebo group (n = 8)** **P-value**
Lactobacillaceae	6.08 (2.90-33.41)	16.40 (0.81-51.34)	0.461
Clostridiaceae	38.26 (10.25-52.29)	3.86 (0.97-28.65)	0.008
Erysipelotrichaceae	3.66 (0.53-13.18)	0.71 (0.11-6.20)	0.008
Eubacteriaceae	1.90 (0.80-3.03)	0.25 (0.05-2.14)	0.039
Streptococcaceae	4.67 (0.61-29.90)	15.35 (5.30-61.93)	0.109
Ruminococcaceae	12.46 (5.88-20.03)	3.07 (0.47-15.01)	0.008
Bacteroidaceae	0.31 (0.00-1.00)	1.23 (0.14-4.01)	0.109
Alcaligenaceae	0.07 (0.00-0.70)	0.15 (0.02-3.18)	0.383
Leuconostocaceae	0.20 (0.06-1.89)	0.05 (0.00-0.30)	0.078
Prevotellaceae	0.40 (0.00-0.89)	0.52 (0.05-23.98)	0.313
Lachnospiraceae	0.54 (0.06-1.99)	0.36 (0.04-0.71)	0.148
Enterobacteriaceae	0.26 (0.02-2.27)	0.07 (0.02-20.01)	0.547
Coriobacteriaceae	1.06 (0.37-1.52)	0.48 (0.05-2.00)	0.461
Fusobacteriaceae	0.00 (0.00-3.45)	0.81 (0.00-28.37)	0.109
Succinivibrionaceae	0.09 (0.00-0.33)	0.07 (0.00-1.02)	0.945
Enterococcaceae	0.09 (0.00-0.60)	0.02 (0.00-0.16)	0.148

### Quantitative real-time PCR

Analysis by qPCR comparing baseline to 2 wk after treatment, demonstrated a statistically significant increase in the abundance of *Lactobacillus* (P = 0.02) and *Bifidobacteria* spp. (P = 0.008) in feces of the synbiotic-fed dogs, which was not observed in the placebo-fed dogs. Fecal abundance of *Enterococcus* spp. was not significant different when comparing these same time points in both groups (Tables 3 and 4).

**Table 3:** Quantitative real-time PCR results expressed as percent (medians and ranges) of microbial genera in the feces of synbiotic-fed dogs at baseline and after 2 wk of treatment

**Genera**	**Synbiotic group (n = 9)** **% at baseline**	**Synbiotic group (n = 9)** **% after 2 wk of treatment**	**Synbiotic group (n = 9)** **P-value**
*Lactobacillus* spp.	3.71 (0.47-20.15)	16.04 (0.42-26.82)	0.02
*Bifidobacterium* spp.	0.02 (0.00-0.12)	0.31 (0.02-1.27)	0.01
*Enterococcus* spp.	0.05 (0.00-0.79)	0.12 (0.03-0.43)	0.95

**Table 4:** Quantitative real-time PCR results expressed as percent (medians and ranges) of microbial genera in the feces of placebo-fed dogs at baseline and after 2 wk of treatment

**Genera**	**Placebo group (n = 8)** **% at baseline**	**Placebo group (n = 8)** **% after 2 wk of treatment**	**Placebo group (n = 8)** **P-value**
*Lactobacillus* spp.	10.07 (0.30-23.18)	10.78 (1.97-28.64)	0.84
*Bifidobacterium* spp.	0.10 (0.00-0.33)	0.09 (0.00-0.95)	0.47
*Enterococcus* spp.	0.02 (0.00-0.40)	0.01 (0.00-0.04)	0.19

## Discussion

Probiotics and prebiotics have been purported to have many beneficial effects on the microbiome of the gastrointestinal tract and immune system of multiple species [11,22-26]. Our results from the 6 wk study period indicate beneficial effects from the use of both a prebiotic and probiotic in combination. The clinical evidence shows that fewer days of diarrhea during a presumed contagious outbreak was observed during wk 5 of treatment. This may be attributed to a microbiome shift of the phylum Firmicutes favoring an increase in *Lactobacillus* spp. The microbiome shift was also represented in the phylum Actinobacteria including an increase in *Bifidobacterium* spp. These shifts may result in production of SCFA such as butyrate and therefore favor enterocyte health and regeneration [15]. Unfortunately, at the end of the 6 wk of treatment, neither *Lactobacillus* or *Bacillus* spp. could be cultured from the synbiotic and therefore, did not appear to colonize the gut effectively. *Enterococcus faecium* SF68 was not included in our bTEFAP microbial analysis; however quantitative real-time PCR results demonstrated a sparse population of *Enterococccus* spp. and no significant increase or decrease in either the synbiotic or placebo-fed groups.

The lack of long-term viability of bacterial strains in the synbiotic formulation was not surprising. A recent evaluation of 25 commercially available products for label accuracy and bacterial content revealed that the overall level of bacterial growth was highly variable, with one product having no viable growth despite its labeling of 14 million CFU/capsule, and another product containing greater than the stated concentration of *Bifidobacterium animalis*[27]*.* Use of a similar synbiotic containing *Bifidobacterium, Enterococcus, Streptococcus,* and four different strains of *Lactobacillus* spp. in dogs showed that only *Enterococcus* and *Streptococcus* counts increased significantly in the feces of dogs and that the *Lactobacillus* and *Bifidobacterium* spp. did not colonize effectively [21].

Our findings suggest significant rises in *Lactobacillus* and *Bifidobacterium* spp, which is contradictory to this previous synbiotic study [21]. In the previous study the synbiotic used a 500 mg capsule containing similar number of probiotic as our synbiotic, but significantly less prebiotic. Dogs in the study were client owned dogs weighing between 10 and 80 lbs. The amount of prebiotic supplied in the diet presumably ranged between approximately 0.05 to 0.4% additional dry matter dietary fiber. This may be enough prebiotic to modestly increase SCFA production [28], but other studies in experimental animals have suggested that to cause significant increases in the fecal microbiome of species such as *Lactobacillus,* that at least 0.5-1% increase in soluble fiber is required [15]. Depending on the daily caloric intake of the dogs in our study (between 1700 and 3200 kilocalories) the prebiotic dose (2.5 grams/day) would translate into 0.5-1% of the dry matter intake which is similar to doses previously used to achieve microbiome changes [15]. This large prebiotic dose leads to the premise that the prebiotic in the diet played a part in the modest microbiome shift in the synbiotic treated dogs, not the probiotic. The microbiome shift being from the prebiotic is further supported with the fact that the only probiotic to survive in the synbiotic preparation was *Enterococcus* which showed no increases and is a very minor part of the microbiome.

Another factor that may have allowed us to observe a shift in the microbiome was our uniform population of dogs being fed similarly across the entire trial as well as the constant exposure to the similar environmental variables. In experimental colony dogs relatively uniform population of microbes in the feces has been observed using methods other than pyrosequencing [29]. This may be different in household companion animals where the microbiome hierarchical clustering shows differences based on environmental variables more so than on supplemental synbiotics provided [21].

A previous study suggested that sled dogs show a more pronounced alteration in the fecal microbiome after 300 miles of racing when compared to a group of field trial Labradors that were in a confined location [30]. Our study controlled for these variables by having identical kenneling, activity, feeding practices, and all dogs traveled to races and trained together; however when examining the dendrogram it is obvious that there is clustering of cases primarily based on time of sampling. Changes observed were in all dogs which included significant decreases in Clostridiaceae, Erysipelotrichaceae and Eubacteriaceae. Whether this shift in colonic microbial flora was due to the addition of a fiber source (soluble or insoluble), or gradual change in environmental variables such as weather, training pattern, exposure to different environments due to travel and/or changes in bedding cannot be determined by our study design. These changes suggest that future studies examining microbiome changes over time due to environmental alterations are warranted.

There was also an apparent synbiotic effect since the majority of synbiotic dogs in the dendrogram samples post-treatment cluster together in one area to the middle left of the dendrogram which is due to an increase in Lactobacillaceae and Streptococcaceae and decreases in Clostridiaceae suggesting that the prebiotic may be the reason for this. Prebiotic in the form of FOS/MOS like the one in our study have shown that acid producing bacterial populations such as Lactobacillacae and Bifidobateriaceae should all increase with an oligosaccharide rich diet which we show trends for [15]. *Bifidobacteria* data were not generated during our pyrosequencing (which is a shortcoming of this technique at the time of analysis); however our quantitative real-time PCR results show a small population in the fecal microbiome that does increase modestly from baseline to 2 wk after treatment in the synbiotic group that was not observed in the placebo group.

There was a large variation in overall abundance of fecal flora represented for certain strains such as *Lactobacillus*, *Enterococcus*, *Streptococcus*, *Ruminococcus*, and *Fusobacteria* spp. This allowed us to examine the microbial families in relation to the total SCFA concentrations as well as each of the SCFA (acetate, butyrate, and propionate) independently. There was a modest correlation between *Lactobacillus* spp. and overall butyrate concentration based on linear regression analysis when examining all dogs in the study. Butyrate has been associated with improved enterocyte health and is a byproduct of the fermentation of fibers; including prebiotics such as inulin-type fructans [31]. Unfortunately, we did not observe an increase in overall SCFA or butyrate concentrations in the feces collected from the synbiotic group. This may be due to a lack of power and the large magnitude of microbiome shift other than just *Lactobacillus* spp., particularly since the synbiotic groups showed a rise in *Lactobacillus* spp. with a decrease in *Rumenococcus spp.* which are both acid producing bacterial populations.

An obvious weakness in our study was our inability to further differentiate the role of each component of the synbiotic and it would have been ideal to have both prebiotic and probiotic alone fed groups. These studies will help differentiate the effects of the prebiotic as the sole agent for inducing alteration in the fecal microbiome which we suggest is the most significant portion of the synbiotic based on our present findings. Additionally, future experiments should incorporate more frequent culturing of the products administered as well as more frequent testing of fecal samples, which will ensure viability of probiotic organisms and allow observation of microbiome shifts over time. Furthermore, the placebo and synbiotic had other constituents such as B vitamins and yeast incorporated, though negligible amounts in general, these components may have influenced the microbiome in some fashion. Most egregious of these differences was the addition of thiamine, riboflavin, niacin and pantothenate to the synbiotics which were not in the placebo. Although we would expect adequate absorption and elimination of these vitamins through renal excretion the amount that could have made it to the colon and its effects on the microbiome cannot be determined. It should also be noted while this was not a cross-over design, all dogs were housed together, exercised identically, and exposed to the same food and environmental variables; therefore the differences between the groups is most likely due to treatment effect, while the environmental variables observed were not expected, making environment a significant factor in host microbiome interactions that warrants future investigation.

From a clinical perspective, the most important observation was that the addition of the placebo or the synbiotic did not cause an increase or decrease in overall fecal consistency over the entire study. This is a pertinent point as this trial used a dose of 5 grams per day which provided well over 10^8^ of each bacterium as well as 2.5 grams of prebiotic accounting for between 0.5 to 1% of dry matter intake for each dog. More significant, we were able to observe a difference in fecal consistency after 5 weeks of treatment during a presumed contagious outbreak of diarrhea in this kennel of sled dogs. Remarkably the synbiotic group showed fewer affected dogs (3/9 synbiotic vs. 4/8 placebo) which was not statistically significant, but more importantly the synbiotic-fed dogs had fewer days of diarrhea than placebo-fed dogs. Though speculative the modest increases in Lactobacillus in the synbiotic group might have led to increased beneficial SCFA at the level of the gastrointestinal mucosa leading to improved enterocyte function during the bout of presumed viral diarrhea leading to hastened recovery.

## Conclusions

The efficacy of probiotics, prebiotics and synbiotics in the veterinary market are not well established when examined in relationship to disease or stress-induced diarrhea. More often the use of prebiotic to ameliorate diarrhea is utilized by veterinary clinicians with a recent increase in probiotic use due to veterinary approved products inundating the market. From a clinical perspective our findings support the use of a synbiotic during contagious diarrhea or during times when relative risk (racing season with extensive kennel-kennel interaction) for transmission of contagious diarrhea is increased to improve the gastrointestinal recovery process.

### Endnotes

^a^Food and Agriculture Organization & World Health Organization (2001) Health and Nutritional Properties of Probiotics in Food Including Powder Milk with Live Lactic Acid Bacteria. Report of a Joint FAO/WHO Expert Consultation on Evaluation of Health and Nutritional Properties of Probiotics in Food Including Powder Milk with Live Lactic Acid Bacteria. Rome: FAO.

^b^Florentero, Candioli Pharma, Rome, Italy.

^c^Ultra 32%, Annamaet Pet foods, Sellersville, PA.

^d^Impact, Annamaet Pet Foods, Sellersville, PA.

^e^Systat Software Inc, San Jose, CA.

## Competing interest

The authors declare that they have no competing interests.

## Authors’ contributions

JWG, KWS and JJW: All participated in the genesis of the hypothesis, data generation, interpretation of the data and generation of the manuscript. KSS and GCF: Participated in the generation and interpretation of the data and manuscript preparation. SL, SED, Dawn AB and Kit B: participated in the generation and interpretation of data. All authors read and approved the final manuscript.

## Supplementary Material

Additional file 1 Commercial feed ingredient lists for feeds utilized in study. Click here for file
