# Supplementary material for: Effects of a synbiotic on fecal quality, short-chain fatty acid concentrations, and the microbiome of healthy sled dogs
Source: BMC Vet Res. 2013 Dec 5;9:246. doi: 10.1186/1746-6148-9-246 (PMC4029452; doi:10.1186/1746-6148-9-246)
Supplement: Additional file 1 — Commercial feed ingredient lists for feeds utilized in study. [file 1746-6148-9-246-S1.docx]

Additional file 1

Ultra 32%, Annamaet Pet Foods: Chicken meal, Chicken, Rice flour, Chicken fat [preserved with natural mixed tocopherols (Vitamin E)], Ground corn, Beet pulp, Corn gluten meal, Wheat flour, Fish meal, Wheat germ meal, Egg product, Oat meal, Brewers yeast, Flax seed meal, Lecithin, Salt, Calcium carbonate, DL-Methionine, Potassium chloride, Ascorbic acid, Lysine, Propionic acid, Vitamin E supplement, d-Calcium pantothenate, Biotin, Niacin, Vitamin A acetate, Riboflavin supplement, Thiamine mononitrate, Pyridoxine hydrochloride, Vitamin B_12_ supplement, Menadione dimethylpyrimidinol bisulfite (source of Vitamin K activity), Citric acid, D-activated animal sterol (source of Vitamin D_3_), Folic acid, Potassium sulfate, Ferrous sulfate, Zinc sulfate, Zinc proteinate, Iron proteinate, Manganese sulfate, Manganese proteinate, Copper sulfate, Copper proteinate, Manganous oxide, Sodium selenite, Copper oxide, Calcium iodate.

Impact, Annamaet Pet Foods: Dried eggs, Chicken, Pork meal, Herring meal, Pork lard (preserved with BHA), Poultry liver, Flax, Coconut oil, Ascorbic acid, Brewers yeast, Potassium chloride, Choline chloride, Vitamin E supplement, Casein, Lecithin, Calcium carbonate, Magnesium oxide, Salt, Ferrous sulfate, Zinc sulfate, Manganese sulfate, Vitamin B_12_ supplement, Copper sulfate, D-Calcium pantothenate, Pyridoxine hydrochloride, Vitamin A acetate, Vitamin D_3_ supplement, Thiamine mononitrate, Calcium iodate.
